# Prevalence and Risk Factors of Functional Constipation According to the Rome Criteria in China: A Systematic Review and Meta-Analysis

**DOI:** 10.3389/fmed.2022.815156

**Published:** 2022-02-16

**Authors:** Zhe Chen, Yingying Peng, Qingyang Shi, Yongjie Chen, Lujia Cao, Jiannan Jia, Chunxiang Liu, Junhua Zhang

**Affiliations:** ^1^Evidence-Based Medicine Center, Tianjin University of Traditional Chinese Medicine, Tianjin, China; ^2^First Teaching Hospital of Tianjin University of Traditional Chinese Medicine, Tianjin, China; ^3^National Clinical Research Center for Chinese Medicine Acupuncture and Moxibustion, Tianjin, China; ^4^Chinese Evidence-Based Medicine Center, West China School of Medicine, Sichuan University, Chengdu, China; ^5^Department of Epidemiology and Statistic, School of Public Health, Tianjin Medical University, Tianjin, China; ^6^Department of Gastroenterology, Tianjin Hospital of Integrated Traditional Chinese and Western Medicine Nankai Hospital, Tianjin, China

**Keywords:** functional constipation, prevalence, Rome criteria, meta-analysis, systematic review

## Abstract

**Background:**

Functional constipation (FC) is a common bowel disorder that prevails worldwide. In China, although a heterogeneous prevalence of constipation is expected, it is currently not demonstrated. In this study, we aimed to evaluate the prevalence and related risk factors of FC in the Chinese population, according to the Rome criteria.

**Methods:**

We searched the PubMed, the Embase, the Cochrane Library, the Web of Science, the China National Knowledge Infrastructure (CNKI), the Wanfang data knowledge service platform, the VIP information resource integration service platform, and the Chinese Biomedical Literature Service System (SinoMed) databases from the inception of database to July 2021. Population-based cross-section studies that enrolled adults with FC, diagnosed by the Rome criteria, were deemed eligible. We summarized the overall prevalence and detected the subgroup effect per the Rome I, Rome II, Rome III, and Rome IV criteria. We used the generalized linear mixed model (GLMM) with a random-effect intercept to pool the prevalence and performed pairwise meta-analyses for prevalence comparisons by risk factors.

**Results:**

We identified 3,213 records through our database search, and 39 studies from China, comprising 1,240,79 participants, met the eligibility criteria for our study. The pooled overall prevalence of FC using the Rome criteria was 8.5% in China. Heterogeneous prevalence was detected within the Rome criteria (Rome II: 10.6%, 95% CI: 7.2–15.4; Rome III: 6.5%, 95% CI: 3.4–12.0; Rome IV: 8.1%, 95% CI: 5.6–11.8). The prevalence increased between 1991 and 2020 (from 5.5% with 95% CI: 3.6–8.2 between 1991 and 2000 to 10.9% with 95% CI: 5.5–20.4 between 2011 and 2020). Higher prevalence was found in women [odds ratio (OR) = 1.53, 95% CI: 1.31–1.78] and the elderly (≥70 years vs. ≤ 29 years: OR = 3.38, 95% CI: 2.16–5.30) than in men and the younger population. A high-fiber diet was associated with lower prevalence (OR = 0.33, 95% CI: 0.15–0.75), whereas irregular bowel habit and inactivity were associated with higher prevalence (OR = 3.64, 95% CI: 2.64–5.03; OR = 1.97, 95% CI: 1.14–3.43). Unhealthy mental states, such as anxiety and depression, and poor sleep quality led to high prevalence (OR = 3.16, 95%C I: 1.96–5.11; OR = 2.74, 95% CI: 1.76–4.26; OR = 2.14, 95% CI: 1.69–2.72, respectively).

**Conclusion:**

Various types of FC prevail in China based on the different Rome criteria, personal characteristics, and habits. The prevalence also increased over the past three decades. The FC should be included under the primary care setting with uniform diagnosis criteria in China.

**Systematic Review Registration:**

http://www.crd.york.ac.uk/PROSPERO, CRD42021277172.

## Introduction

Constipation, a common symptom among the general adult population, is defined as a bowel disturbance characterized by hard or dry stools, infrequent bowel movements, and incomplete bowel evacuation (1–3). Constipation creates a negative impact on a patient's quality of life; the patients mainly seek help from gastrointestinal experts and medical institutions (2). Functional constipation (FC) is a functional gastrointestinal disorder (FGID) that presents without organic lesions and physiological abnormalities and is diagnosed as per the Rome criteria (4). Compared to the non-uniform criteria with self-reported definitions, the Rome criteria improved the accuracy of the diagnosis of constipation and helped estimate the prevalence of FC (5).

The Rome criteria followed evidence-based medicine and provided clear decision-making standards and definitions for clinical practice, which were recognized and generalized by clinical researchers (6, 7). With accumulating evidence in etiology, epidemiology, and pathophysiology, the diagnostic criteria for FC were modified and optimized, leading to classification into Rome I, Rome II, Rome III, and Rome IV (1, 8, 9) criteria. Among the various FGIDs, the FC has become a research hotspot with its high prevalence among the general population (3). Although there were some discrepancies in the iterations of the Rome criteria, the pooled prevalence of FC at 6.8–15.3% remained high (10, 11).

The FC places a considerable economic burden on health care resources (2, 12, 13). Patients who experience persistent constipation are more susceptible to mental health challenges and are associated with increased all-cause mortality (14). Risk factors of constipation are related to the living environment, dietary habits, and unhealthy behaviors (15–17). A large fraction of patients with FC tends to self-medicate, which could affect the optimal timing of treatment and exacerbate the disease (13, 17). Constipation-related risk factors can cause severe comorbid medical problems; therefore, detailed studies are crucial to improve the current status (17–19).

With rapid economic development in China, the prevalence of constipation has increased in China and has now become a serious public health issue (20). Although multiple cross-sectional studies and related systematic reviews have reported the estimated prevalence of constipation in China, they have used self-defining criteria (21–23). The Rome criteria are widely relevant for diagnosing gastrointestinal disorders, and cross-sectional studies of FC diagnosed according to the Rome criteria have increased recently in China. Clinical evidence on the prevalence of FC diagnosed according to the Rome criteria and constipation-related risk factors have been lacking in China, leading to an incomplete understanding of constipation, which was not conducive to effective clinical practice.

An updated systematic review on the global prevalence of FC suggests that its prevalence with uniform symptom-based criteria still varies considerably among countries (11). Thus, the precise diagnosis of FC based on the Rome criteria is necessary to reduce the geographical variability. There is a lack of evidence to support constipation-related risk factors; therefore, we did a systematic review and meta-analysis to elucidate the reasons for pooled prevalence and related risk factors of FC using the Rome criteria among the general population in China.

## Methods

The protocol of this systematic review (register number: CRD 42021277172) was registered in PROSPERO (www.crd.york.ac.uk/PROSPERO). We carried out this systematic review according to the Preferred Reporting Items for Systematic Reviews and Meta-analyses (PRISMA) guidelines (24).

### Search Strategy

In this systematic review and meta-analysis, we searched the main Chinese electronic databases, the China National Knowledge Infrastructure (CNKI), the Wanfang Data Knowledge Service Platform, the VIP information resource integration service platform, the Chinese Biomedical Literature Service System (SinoMed), the English electronic databases, the PubMed, the Embase, the Cochrane Library, and the Web of Science, from their inception to July 2021, to identify the population-based cross-sectional studies that reported the prevalence and related risk factors of FC diagnosed according to the Rome criteria in adult populations in China. The search strategy was applied by two independent investigators (ZC and YP), and a third researcher (CL) was responsible for resolving any disagreement. The search terms were a combination of MeSH terms and free text, as discussed by the specialist consensus on the search terms, which included “constipation,” “chronic constipation,” “functional constipation,” “idiopathic constipation,” “bowel disorders,” “functional gastrointestinal disorders,” etc. Additionally, the following terms related to the Rome criteria were also used: “Rome I,” “Rome 1,” “Rome II,” “Rome 2,” “Rome III,” “Rome 3,” “Rome IV,” and “Rome 4.” We also manually retrieved data from the included cross-sectional surveys to supplement the literature and ensure a comprehensive search. There were no language restrictions. We translated the literature published in languages other than Chinese to understand it better. More detailed information of search strategy is reported in Supplementary File 1.

### Eligibility Criteria

We included studies if the prevalence and related risk factors of FC were reported in the general population or community population. To be eligible, recruitment should have been conducted in China, including Mainland China and Taiwan Province, to recruit the eligible healthy adult Chinese (age older than 18 years). Further, studies that recruited more than 100 participants were included. We also included studies that reported the prevalence of functional gastrointestinal disorders or bowel disorders or incidence of FC diagnosed using the Rome criteria. All the included studies were conducted on humans.

Ineligible participants included college students, special professionals (coal miners, soldiers, medical staff, etc.), people undergoing health checks in primary care clinics due to illness or poor physical condition, participants under 18 years, and pregnant women. We also excluded studies where the diagnostic criteria of FC were unclear and self-defined and excluded studies that focused on irritable bowel syndrome with constipation (IBS-C).

### Data Extraction

The title, abstract, and the full text of eligible articles were independently screened by two investigators (ZC and YP). All data from the eligible articles were extracted according to the prespecified eligibility criteria and checked by a third investigator (QS) for accuracy and completeness. When data from multiple studies were obtained for the same group of participants, we chose the studies with more detailed information. We contacted the authors of papers with ambiguous information or missing data. Any inconsistencies were resolved and discussed until a panel of researchers (CL and JZ) reached a consensus.

The following data were extracted: study characteristics (study author, publication year, study year, research type, prevalence rate, response rate, province, questionnaire method, subtype under the Rome criteria), basic information of participants [gender, median age, age band, educational level, socioeconomic status, and body mass index (BMI)]. Data extracted from related risk factors included the following: dietary habits (high-fiber diet, meat, drinking water situation, and drinking water time), living habits (physical activity and bowel habit), working conditions (labor types, working pressure, working status, and constipation management), related diseases (abnormal mental state: anxiety, depression, and poor sleep quality; circulatory system diseases: coronary heart disease and hypertension; digestive system diseases: aerophagia, biliary tract disease, bloating, chronic gastritis, dyspepsia, gastroesophageal reflux, intestinal polyp, irritable bowel syndrome, and steatohepatitis; endocrine system diseases: diabetes, hyperhomocysteinemia, hyperlipidemia, hyperuricemia, and thyroid disease), unhealthy behavior (smoking and alcohol drinking), and self-management behavior.

### Statistical Analysis

We used the generalized linear mixed model (GLMM) with the random-effect method and fixed-effect method to pool the single group proportions for the included studies. The proportion was modeled by a logit transformation in GLMM. The between-study variance was estimated using the DerSimonian-Laird estimator with corresponding *I*^2^ and Q statistic for quantifying and testing the heterogeneity. The funnel plot with Egger's and Begg's test quantified the publication bias; we then used trim and fill analysis, if the bias was detected.

Several subgroup analyses were introduced, including the province, Rome criteria, sampling year, sampling method, questionnaire method, gastrointestinal disease type, and check if the questionnaires were validated or not.

Several comparisons of prevalence were performed according to the following factors: (1) general information, such as sex, age, education, socioeconomic status, and BMI; (2) diet, including high-fiber or not, meat intake or not, and water intake is regular or not; (3) lifestyle habit, including physical activity, work habits, and bowel movement, (4) related diseases, including mental status, cardiovascular diseases, gastrointestinal diseases, and endocrine diseases; and (5) smoking and drinking habit. These parameters were compared using the OR with 95% CI from pairwise meta-analyses using the Mantel-Haenszel pooling method.

All analyses were done using R 4.0.5 with a meta package.

## Results

**Figure 1:**
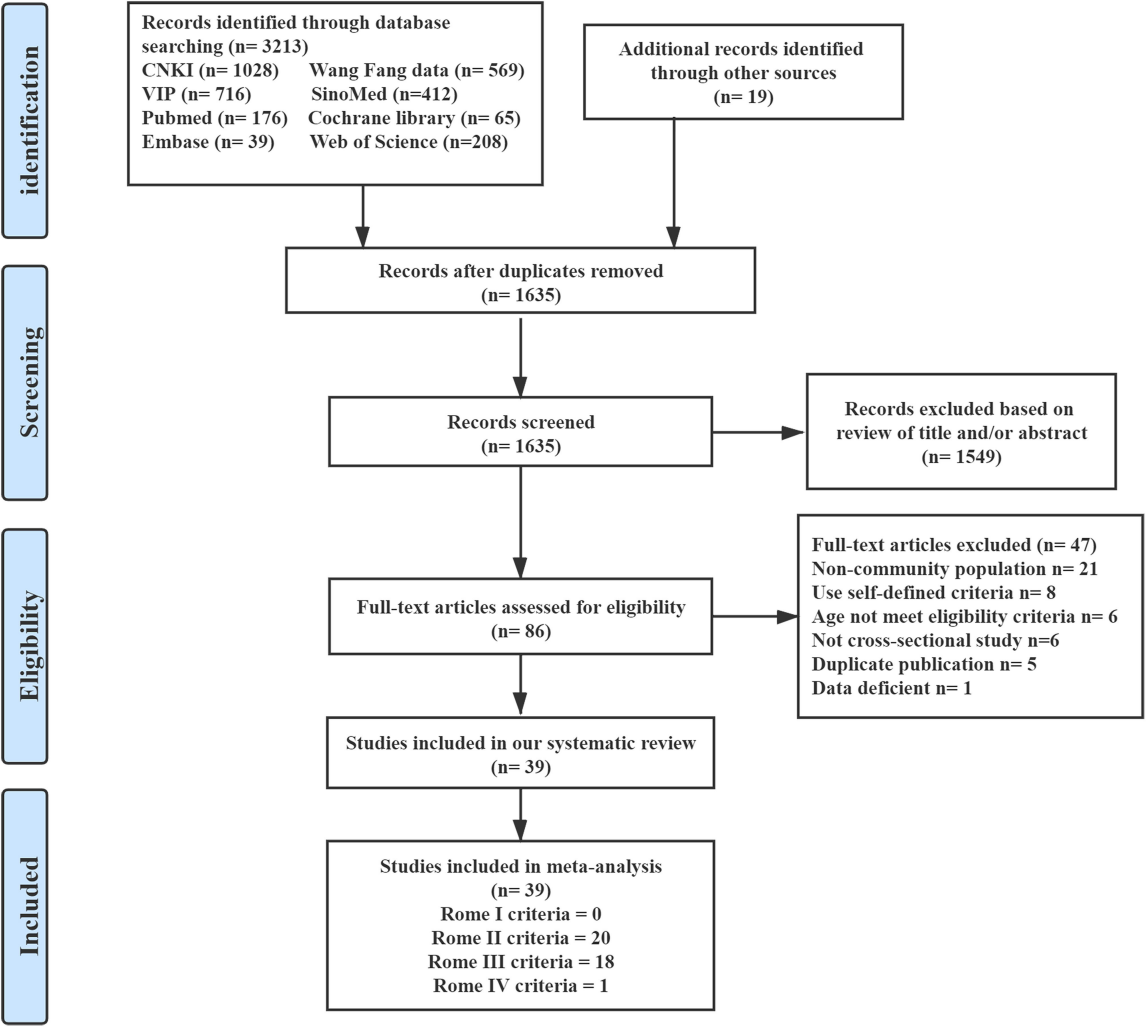
Summary of evidence survey and selection. CNKI, the China National Knowledge Infrastructure; SinoMed, the Chinese Biomedical Literature Service System.

We identified 3,213 citations by searching the databases and found 19 additional studies on running a manual search. Of these, we identified 39 studies that reported the prevalence of FC among the general adult populations, after being appraised and assessed for eligibility. The results of the search are summarized in Figure 1. We included data from 39 eligible cross-sectional studies (25–63), containing 1,240,79 healthy adults recruited in China. Detailed characteristics of all the included studies are reported in Supplementary Table 1. The lowest prevalence (0.25%) and the highest prevalence (38.82%) were reported in two studies (47, 57) that used Rome III criteria in the interview-administered questionnaire (Supplementary Table 1).

### Prevalence of FC

#### Prevalence of FC Stratified by the Rome Criteria

**Figure 2:**
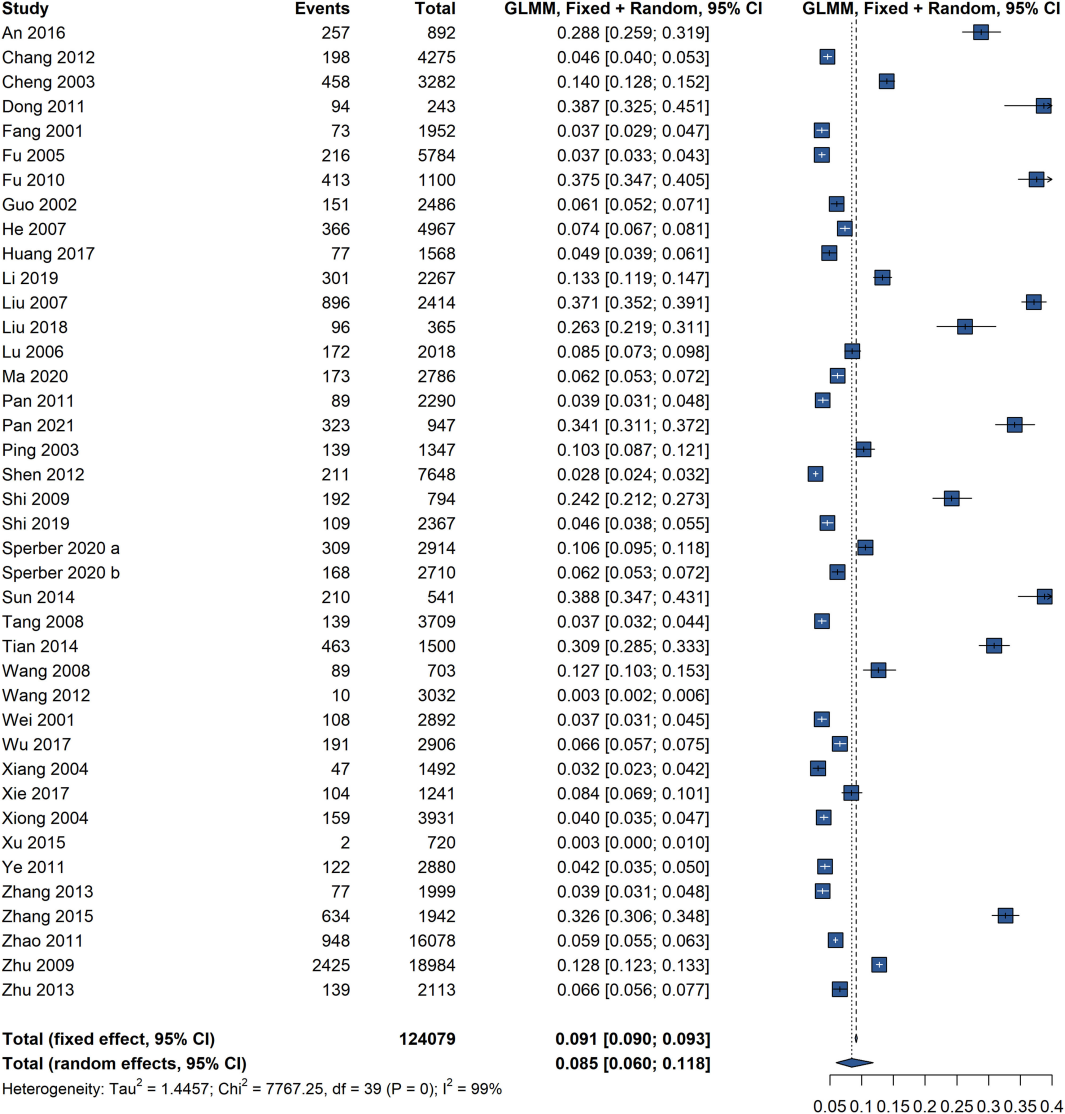
Forest plot of prevalence of functional constipation according to the Rome criteria.

Based on 39 studies involving 1,240,79 participants in China, the overall pooled prevalence of FC using the Rome diagnostic criteria was 8.5% (95% CI: 6.0–11.8), with significant heterogeneity (*I*^2^ = 99%, *P* = 0) (Figure 2). The pooled prevalence of FC in China, according to various Rome criteria used to diagnose FC, is shown in Table 1. There was no pooled prevalence of FC was found according to the Rome I criteria. Twenty studies, eighteen studies, and only one study used the Rome II criteria, Rome III criteria, and Rome IV criteria, respectively. The difference in the pooled prevalence of FC according to the Rome II criteria was higher (10.6%, 95% CI: 7.2–15.4) than those according to the Rome III (6.5%, 95% CI: 3.4–12.0) and Rome IV criteria (8.1%, 95% CI: 5.6–11.8), with significant heterogeneity.

**Table 1:** Pooled prevalence of functional constipation according to the criteria used to define its occurrence, year of study, method of questionnaire administration, validation status of questionnaire, methods of sampling, and research based on disease types.

	**Number of studies**	**Number of participants**	**Pooled prevalence (95% confidence interval)**	***I^**2**^***	***p*-value for ***χ^2^*****
**CRITERIA USED TO DEFINE ITS PRESENCE**					
Rome I	NA	NA	NA	-	-
Rome II	20	77,140	10.6% (7.2–15.4)	100%	0
Rome III	18	41,315	6.5% (3.4–12.0)	100%	0
Rome IV	1	5,624	8.1% (5.6–11.8)	97%	<0.01
**STUDY YEAR**					
Between 1991 and 2000	4	8,677	5.5% (3.6–8.2)	97%	<0.01
Between 2001 and 2010	20	89,310	8.5% (5.7–12.4)	100%	0
Between 2011 and 2020	12	23,130	10.9% (5.5–20.4)	99%	0
**METHOD OF QUESTIONNAIRE ADMINISTRATION**					
Interview-administered questionnaire	27	68,996	7.3% (4.6–11.3)	99%	0
Self-administered questionnaire	5	43,296	11.9% (6.3–21.5)	100%	0
Unclear administered questionnaire	8	11,787	11.0% (5.5–20.7)	99%	<0.01
**VALIDATION STATUS OF QUESTIONNAIRE**					
Validated	3	24,984	8.6% (6.0–12.3)	99%	<0.01
Not validated	36	99,095	8.4% (5.7–12.2)	100%	0
**SAMPLING METHODS**					
Stratify and random sampling	15	76,740	6.7% (4.8–9.2)	99%	0
Random sampling only	12	22,473	13.1% (7.2–22.5)	99%	0
Convenience sampling	12	24,866	7.2% (3.1–15.5)	99%	0
**DISEASE TYPES OF RESEARCH**					
Constipation only	27	75,452	12.1% (8.4–17.1)	100%	0
Constipation and other gastrointestinal disorders	12	48,627	3.9% (2.2–6.7)	97%	<0.01

*NA, not applicable*.

#### Prevalence of FC Stratified by Study Setting

All information of prevalence that was stratified by study settings is shown in Table 1. Four studies on the prevalence of FC were conducted between 1991 and 2000 (5.5%, 95% CI: 3.6–8.2), 20 studies were conducted between 2001 and 2010 (8.5%, 95%CI: 5.7–12.4), and 12 studies were conducted between 2011 and 2020 (10.9%, 95% CI: 5.5–20.4). The pooled prevalence of FC was higher in the studies conducted between 2011 and 2020 than in the other year bands.

Most studies reported the method of questionnaire administration, with 27 studies using the interview-administered questionnaire (7.3%, 95% CI: 4.6–11.3), and five studies using the self-administered questionnaire (11.9%, 95% CI: 6.3–21.5). There were eight studies reporting the method of questionnaire administration with unclear data (11.0%, 95% CI: 5.5–20.7).

Three of all the included studies reported that they used a validated questionnaire, and 36 studies had not reported the validation status of the questionnaire. Compared to the validated questionnaire (8.6%, 95% CI: 6.0–12.3), the prevalence of FC was almost approximated in studies that used the non-validated questionnaire (8.4%, 95% CI: 5.7–12.2).

We summarized three sampling methods in this meta-analysis, with 15 studies using stratified and random sampling (6.7%, 95% CI: 4.8–9.2), 12 studies using random sampling only (13.1%, 95% CI: 7.2–22.5), and 12 studies using the convenience sampling (7.2%, 95% CI: 3.1–15.5).

For all eligible studies, we divided the research on various disease types into two categories: 27 studies that only focused on constipation with an unexpectedly high prevalence of FC (12.1%, 95% CI: 8.4–17.1) were compared with 12 studies that focused on constipation and other gastrointestinal disorders (3.9%, 95% CI: 2.2–6.7).

The pooled prevalence of individual provinces in China, according to the Rome criteria, is shown in Table 2. There was significant heterogeneity between most studies, and the prevalence of FC was the lowest in the Henan province at 1.1% (95% CI: 0.2–6.1) and the highest in the Jilin province at 38.7% (95% CI: 32.8–45.0) and the Ningxia Hui Autonomous Region at 38.8% (95% CI: 34.8–43.0) in China. The heterogeneity in the prevalence of FC by geographical variation was significant in these analyses on the province.

**Table 2:** Pooled prevalence of functional constipation according to the geographic regions in China.

**Geographic regions**	**Province**	**Number of studies**	**Number of participants**	**Pooled prevalence (95% confidence interval)**	***I^**2**^***	***p*-value for ***χ^2^*****
North China	Beijing Municipality	4	22,458	8.6% (3.5–19.6)	100%	<0.01
	Hebei Province	4	5,751	22.0% (10.8–39.6)	99%	<0.01
Northeast China	Jilin Province	1	243	38.7% (32.8–45.0)	NA	NA
Northwest China	Shaanxi Province	2	16,798	1.4% (0.1–11.9)	95%	<0.01
	Xinjiang Uyghur Autonomous Region	2	794	29.0% (22.6–36.3)	95%	<0.01
	Ningxia Hui Autonomous Region	1	541	38.8% (34.8–43.0)	NA	NA
East China	Shanghai Province	6	30,477	5.6% (3.9–8.0)	97%	<0.01
	Zhejiang Province	4	6,690	11.0% (5.1–21.8)	99%	<0.01
	Anhui Province	2	6,589	4.0% (3.5–4.5)	2%	0.31
	Jiangsu Province	1	1,999	3.9% (3.1–4.8)	NA	NA
South Central China	Guangdong Province	4	24,248	5.5% (3.7–8.1)	97%	<0.01
	Henan Province	2	8,816	1.1% (0.2–6.1)	98%	<0.01
	Hubei Province	1	16,078	5.9% (5.5–6.3)	NA	NA
Southwest China	Chongqing Municipality	1	1,492	3.2% (2.4–4.2)	NA	NA
	Sichuan Province	1	4,967	7.4% (6.7–8.1)	NA	NA
Hong Kong, Macao and Taiwan regions of China	Taiwan	2	6,293	6.3% (4.1–9.5)	97%	<0.01
	Hong Kong	1	3,282	14.0% (12.8–15.2)	NA	NA

*NA, not applicable, too few studies to assess heterogeneity*.

#### Prevalence of FC Stratified by Participant Characteristics

The characteristic-specific prevalence estimates of FC are shown in Table 3. In the subgroup analyses by sex, the pooled prevalence was higher in women (10.4%, 95% CI: 7.5–14.3) than in men (7.0%, 95% CI: 4.6–10.7); the differences were significant (OR = 1.53, 95% CI: 1.31–1.78). The pooled prevalence of FC increased with age; it was significant in patients older than 50 years than in those younger than 29 years. Twenty studies provided data of the older age, 50–69 years [8.0% (95% CI: 5.4–11.7); OR =1.41, 95% CI: 1.08–1.85] and 15 studies provided data of people older than 70 years [14.9% (95%C I: 10.0–21.6); OR = 3.38, (95% CI: 2.16–5.30)]. There were no significant differences between educational levels, and the pooled prevalence of FC was slightly higher in patients with only primary education than in those with secondary and college degree. The prevalence of FC also varied with a slight change in the socioeconomic status with specific estimates, and there were no significant differences between patients of lower or medium and higher socioeconomic status. The estimated relationship between the prevalence of FC and BMI was not significantly related and presented with a slight upward trend as BMI decreased.

**Table 3:** Pooled prevalence of functional constipation according to sex, age band, educational level, socioeconomic status, and body mass index (BMI).

	**Number of studies**	**Number of participants**	**Pooled prevalence (95% confidence interval)**	**Odds ratio (95% confidence interval)**	***I^**2**^***	***p*-value for ***χ^2^*****
**Sex**
Male	25	39,408	7.0% (4.6–10.7)	1	-	-
Female	25	40,028	10.4% (7.5–14.3)	1.53 (1.31–1.78)	84%	<0.01
**Age band**
≤ 29 years	13	15,545	4.0% (2.7–5.8)	1	-	-
30–49 years	14	31,591	4.9% (3.3–7.2)	1.09 (0.93–1.29)	62%	<0.01
50–69 years	20	23,975	8.0% (5.4–11.7)	1.41 (1.08–1.85)	83%	<0.01
≥70 years	15	11,614	14.9% (10.0–21.6)	3.38 (2.16–5.30)	87%	<0.01
**Educational level**
Primary	15	14,953	12.3% (6.9–21.0)	1	-	-
Secondary	14	27,040	11.0% (6.4–18.5)	0.87 (0.74, 1.03)	69%	<0.01
College graduate	15	12,523	9.9% (6.1–15.7)	0.97 (0.72, 1.31)	82%	<0.01
**Socioeconomic status**
High	4	1,953	8.1% (5.4–12.1)	1	-	-
Medium	4	9,386	10.1% (6.5–15.4)	1.30 (0.76–2.22)	0.87	<0.01
Low	4	11,770	10.0% (4.8–19.8)	1.28 (0.58–2.84)	-	-
**Body mass index**
**≤24.9 and** **≥25**
≤ 24.9	5	20,174	4.3% (3.5–5.4)	1	-	-
≥25	4	2,232	4.2% (2.7–6.5)	1.09 (0.67–1.76)	73%	0.01
**≤18.5 and 18.5–24.9**
≤ 18.5	3	2365	5.3% (2.9–9.5)	1	-	-
18.5–24.9	3	15036	4.3% (3.2–5.9)	0.79 (0.51–1.23)	73%	0.02

### Comparison of Prevalence of FC by Risk Factors

#### Lifestyle

Dietary habits are risk factors for FC; therefore, we analyzed the following related aspects (Supplementary Table 2). There were significant differences between patients on diets with high fiber (OR = 0.33, 95% CI: 0.15–0.75), fruits (OR = 0.51, 95% CI: 0.28–0.92), vegetables (OR = 0.38, 95% CI: 0.29–0.51), and coarse grains (OR = 0.44, 95% CI: 0.35–0.57) and those on diets without them. Compared to those who did not consume meat (8.9%, 95% CI: 3.5–20.8), the prevalence of FC in patients who consumed meat (22.6%, 95% CI: 9.0–46.4) was significantly different (OR = 2.92, 95% C I: 2.17–3.93) with no significant heterogeneity between studies (*I*^2^ = 0 %, *P* = 0.81). We found that the OR for FC in patients whose drinking water intake was moderate or high was 0.34 (95% CI: 0.10–1.14) and 0.35 (95% CI: 0.14–0.87), respectively, compared to that in patients whose drinking water intake was low. There was a significant difference in drinking water in the morning vs. other times (OR = 0.71, 95% CI: 0.57–0.90).

Other lifestyles that were reported were analyzed as follows and are also summarized in Supplementary Table 2. The pooled prevalence of FC was higher in patients with a lifestyle that involved infrequent physical activity than in those whose lifestyle involved frequent physical activity [16.7% (95% CI: 8.8–29.3) vs. 9.1% (95% CI: 5.5–14.6); OR= 1.97 (95% CI: 1.14–3.43)]; prevalence was also higher in patients with an irregular bowel habit than in those with a regular bowel habit [41.6% (95% CI: 24.5–61.1) vs 15.7% (95% CI: 7.7–29.5); OR= 3.64 (95% CI: 2.64–5.03)]. We found that patients involved in manual labor were less susceptible to FC with no significant differences. There was a significant increase in the pooled prevalence of FC in patients subjected to high working pressures than in those subjected to low working pressure [35.6% (95% CI: 29.6–42.1) vs. 11.9% (95%CI: 7.5–18.3); OR= 4.09 (95%CI: 2.3–7.29)]. There were no significant differences in the pooled prevalence of FC in patients who were working or retired vs. those who were not working. In terms of smoking and alcohol intake, we found that there were no significant differences in patients who used to or currently smoke or drink vs. those who never smoked and drank.

#### Related Disease

We assessed the effects of related disease with FC in this meta-analysis and the data are shown in Supplementary Table 3. For abnormal mental state, the meta-analysis showed that significant differences existed in the pooled prevalence for anxiety [20.3% (95% CI: 11.1–34.2) vs. 6.9% (95% CI: 4.5–10.1); OR = 3.16 (95% CI: 1.96–5.11)], depression [20.8% (95% CI: 11.3–35.2) vs. 8.1% (95% CI: 4.9–13.3); OR = 2.74 (95% CI: 1.76–4.26)], poor sleep quality [33.8% (95% CI: 29.2–38.6) vs. (18.2% (95%CI: 12.9–24.9); OR = 2.14 (95%CI: 1.69–2.72)] participants with and without FC, and the prevalence in people with FC was found to be higher than in those without FC. We found that the pooled prevalence of dyspepsia and gastroesophageal reflux was higher in participants with FC than in those without FC, with OR 6.00 (95% CI: 1.48–24.27) for dyspepsia and OR 2.69 (95% CI: 1.56–4.66) for gastroesophageal reflux. For endocrine system diseases, the pooled prevalence of diabetes in those with FC and without FC were 13.3% (9.2–18.8) and 9.5% (95%CI: 5.6–15.8), respectively, with OR 1.61 (95% CI: 1.09–2.38). There were no significant differences in the pooled prevalence of coronary heart disease, hypertension, biliary tract disease, and hyperlipidemia in participants with FC vs. participants without FC.

#### Self-Management Behavior

The percentage of various self-management behavior of patients with FC for improving constipation were detected with differences. Among those with FC, the percentage of change in diets was the highest in 40.7% of patients (95%CI: 29.9–52.6), followed by self-medication behavior in 23.8% (95% CI: 13.9–37.5), no changes in 23.4% (95%CI: 14.8–34.8), and health-seeking behavior in 17.2% (95%CI: 11.5–24.8) of patients (Supplementary Table 4).

#### Publication Bias Assessment

We found no significant publication bias for the overall pooled prevalence of FC by Egger test (*P* = 0.1054) and Begg's test (*P* = 0.2083). The funnel plot is shown in Supplementary Figure 1.

## Discussion

### Principal Findings

This is the first systematic review and meta-analysis to compile the prevalence data for FC diagnosed according to the Rome criteria from 39 population-based cross-sectional studies, with 124,079 participants. The pooled prevalence of FC across all eligible studies in China was 8.5%, and pooled prevalence in FC diagnosed within the Rome II criteria was 10.6% which was higher than 6.5% in the Rome III criteria. We have demonstrated that the pooled prevalence of FC varied markedly among the provinces in China, at 1.1–38.8%, using the Rome criteria. The pooled prevalence of FC showed a steadily increasing trend between 1991 and 2020, with the highest prevalence (10.9%) observed in studies conducted between 2011 and 2020. Compared with self-administered and unclear questionnaires at 11.9% and 11.0%, the pooled prevalence of FC using the interview-administered questionnaire was higher at 7.3%. Our results suggested that the validation status of the questionnaire used had no significant impact on the prevalence of FC, but the sampling methods and various disease types of research influenced the prevalence. Furthermore, there were strong associations between the pooled prevalence of FC, age bands, and sex. Meta-analysis results suggest that the higher prevalence of FC was more common among women and the elderly, and the ORs were high in women and patients older than 50 years. Results of our study showed that the pooled prevalence of FC was not significantly different with changes in educational level, socioeconomic status, and BMI.

We identified the potential benefits of a high-fiber diet (fruit, vegetable, and coarse grains) in reducing the risk of FC. We further present evidence that high meat intake was a significant risk factor for FC, with an increased OR of 2.92. The pooled prevalence of FC was strongly associated with dietary habits in our findings. The prevalence of FC was more commonly linked to infrequent physical activity, irregular bowel habits, and high working pressure, and there was no association between the pooled prevalence of FC and working status, labor types, smoking, and alcohol intake. We found that the FC was strongly associated with the abnormal mental state (anxiety, depression, and poor sleep quality), dyspepsia, gastroesophageal reflux, and diabetes, and not related to coronary heart disease, hypertension, biliary tract disease, and hyperlipidemia. For self-management behavior in those with FC in China, they have changed in the diet by themselves from 2-fold to 3-fold greater than self-medicine behavior or health-seeking behavior.

### Strengths and Limitations of the Study

To the best of our knowledge, this is the first systematic review and meta-analysis to compile the pooled prevalence of FC diagnosed according to the Rome criteria in general adult populations in China. Some strengths of our study are that we used more granular data based on the Rome criteria for FC than that used in previous studies that reported non-uniformity in the diagnostic criteria and identified all available literature using rigorous methodology. The literature search was conducted independently by two investigators to judge all potentially eligible studies, ensure comprehensive and rigorous extraction of data, and facilitate consensus and discussion by a panel of researchers, if necessary. We used the GLMM method to pool the data, as this may provide a more precise estimation than other methods. We used information gathered from trial settings and the Rome criteria to estimate the pooled prevalence of FC and explored all significant risk factors: constipation-related lifestyle, disease, and self-management behavior. This study focused on the geographic region and the characteristics of the target population, and our results are fully applicable to Chinese individuals living in China.

There were several limitations in our study. Although we analyzed all related risk factors reported in the included studies, there was a deficiency of information on other potential factors that might be strongly associated with FC, because of the limitations in those studies. Due to unavailable data in a limited number of studies, some subgroup analyses could not be conducted. Furthermore, only one study reported the prevalence of FC diagnosed according to the Rome IV criteria with the interview-administered and self-administered questionnaires. Since Rome IV was a new criterion for FC, there was a paucity of data with respect to studies for FC diagnosed with the Rome IV criteria in China. Owing to the lack of information in several provinces of China, the real prevalence of FC in these provinces was not precisely reflected, further affecting the accuracy of pooled prevalence. The high heterogeneity may be relevant in the differences among the related factors in individual studies, including study designs, different diagnostic criteria, and target populations. These limitations can be resolved through cross-sectional studies conducted in the future, with efforts made to interpret high-quality data. Another limitation of this study might be the low number of available studies and wide confidence intervals on possible risk factors.

### Relation and Comparison With Other Studies

A previous and another updated systematic review were published in 2011 and 2021 by the same research team, which reported the global prevalence of FC as 14 and 15.3%, respectively (10, 11). Due to environmental, ethnic, dietary, or cultural differences between various countries, the global prevalence of FC varied, as a uniform symptom-based criteria (11). The estimated prevalence that we calculated is lower than that reported previously; this can be attributed to the differences in study locations. In this meta-analysis, we only focused on the prevalence of FC using all definitions of the Rome criteria in the Chinese population, to minimize the influence of geographical differences on the results and improve accuracy in the prevalence estimates in China. Our results also supported the higher prevalence of FC in women than in men. We observed that the pooled prevalence of FC was higher in patients of increasing age, which is in disagreement with the new updated systematic review (11). A previous meta-analysis concluded that the pooled prevalence of FC, according to different diagnostic criteria across various populations in China, at 8.2% was consistent with our results, according to the Rome criteria, at 8.5% (22). Previous systematic reviews have not identified and assessed the constipation-related risk factors in a particular geographical area (10, 11, 22).

### Implications for Research and Clinical Practice

Findings from this study have important implications for the health policy and clinical practice in China. This meta-analysis showed that people in China commonly experienced constipation. Based on our results, we suggest that future studies should comprehensively consider the risk factors for FC, using the Rome criteria. Patients sought improved quality of life and alleviated the symptoms of constipation by relying on self-medication and consultation, which may increase the burden on public health (12, 64). Furthermore, educating the general public is imperative to enhance self-management, and relevant patient guidelines will be urgently established to improve this situation. We found an association between depression and anxiety and FC; patients on antipsychotic medications may be predisposed to low sensitivity to gastrointestinal symptoms and further instill inaccuracies in the prevalence of FC (65). Our results were consistent with those of previous studies: dietary habits and physical activity were risk factors for FC, but the evidence behind the association of potential diseases with FC is still lacking and remains to be validated (11, 66). Consequently, more studies are needed to investigate and validate those risk factors of FC in China. The implications of those studies also will help provide worldwide health services. The pooled prevalence of FC has a significant gap between studies that focus on FC and other gastrointestinal disorders and studies that focus only on FC. Studies that only focus on constipation should be investigated in future research to improve its accuracy.

## Conclusion

Through this systematic review and meta-analysis, we have demonstrated that the pooled prevalence of FC was 8.5% in China, which varied from 6.5% obtained with Rome III criteria and 10.6% with the Rome II criteria. The FC was more common in women and the elderly. The prevalence varied significantly in different provinces and it had a steadily increasing trend between 1991 and 2020. Based on the support from ample data and evidence, a high-fiber diet, regular bowel movements, and physical activity significantly affect FC. Furthermore, our results confirmed that anxiety, depression, and poor sleep quality were strongly associated with constipation. More FC-related risk factors need to be investigated in the future. Although some variables are unclear, these data can help lay down health policy and improve clinical practices in China to reduce the burden on public health.

## Data Availability Statement

The original contributions presented in the study are included in the article/Supplementary Material, further inquiries can be directed to the corresponding authors.

## Author Contributions

ZC and CL conceived the writing ideas of this manuscript and designed this study. ZC and YP wrote the initial draft of this manuscript and conducted the data analyses. ZC, YP, and QS completed the literature retrieval and data extraction. YC, JJ, LC, CL, and JZ instructed and reviewed the manuscript at any stage of this study. All authors participated in the study and revised and approved this manuscript.

## Funding

This study was supported by the Young Qihuang Scholar Support Project of National Administration of Traditional Chinese Medicine and Special Support Plan for Talent Development of Tianjin—Young Top Talent Project (No. 201504).

## Conflict of Interest

The authors declare that the research was conducted in the absence of any commercial or financial relationships that could be construed as a potential conflict of interest.

## Publisher's Note

All claims expressed in this article are solely those of the authors and do not necessarily represent those of their affiliated organizations, or those of the publisher, the editors and the reviewers. Any product that may be evaluated in this article, or claim that may be made by its manufacturer, is not guaranteed or endorsed by the publisher.
