# Supplementary material for: Prevalence and Risk Factors of Functional Constipation According to the Rome Criteria in China: A Systematic Review and Meta-Analysis
Source: Front Med (Lausanne). 2022 Feb 16;9:815156. doi: 10.3389/fmed.2022.815156 (PMC8889120; doi:10.3389/fmed.2022.815156)
Supplement: Supplementary file 1 [file Data_Sheet_1.DOCX]

**Supplementary materials**

**Supplementary Figure, Tables and File**

[**Supplementary Figure 1 The funnel plot of overall pooled prevalence of functional constipation 2**](#_Toc87560458)

[**Supplementary Table 1 Characteristics of included studies 3**](#_Toc87560459)

[**Supplementary Table 2 Pooled prevalence of functional constipation according to dietary habits, living habits, working conditions, and unhealthy behavior 6**](#_Toc87560460)

[**Supplementary Table 3 Pooled prevalence of functional constipation according to related diseases 9**](#_Toc87560461)

[**Supplementary Table 4 Percentage of functional constipation according to self-management behavior 11**](#_Toc87560462)

[**Supplementary File 1 The Detailed Search Strategy 12**](#_Toc87560463)

# Supplementary Figure 1 The funnel plot of overall pooled prevalence of functional constipation


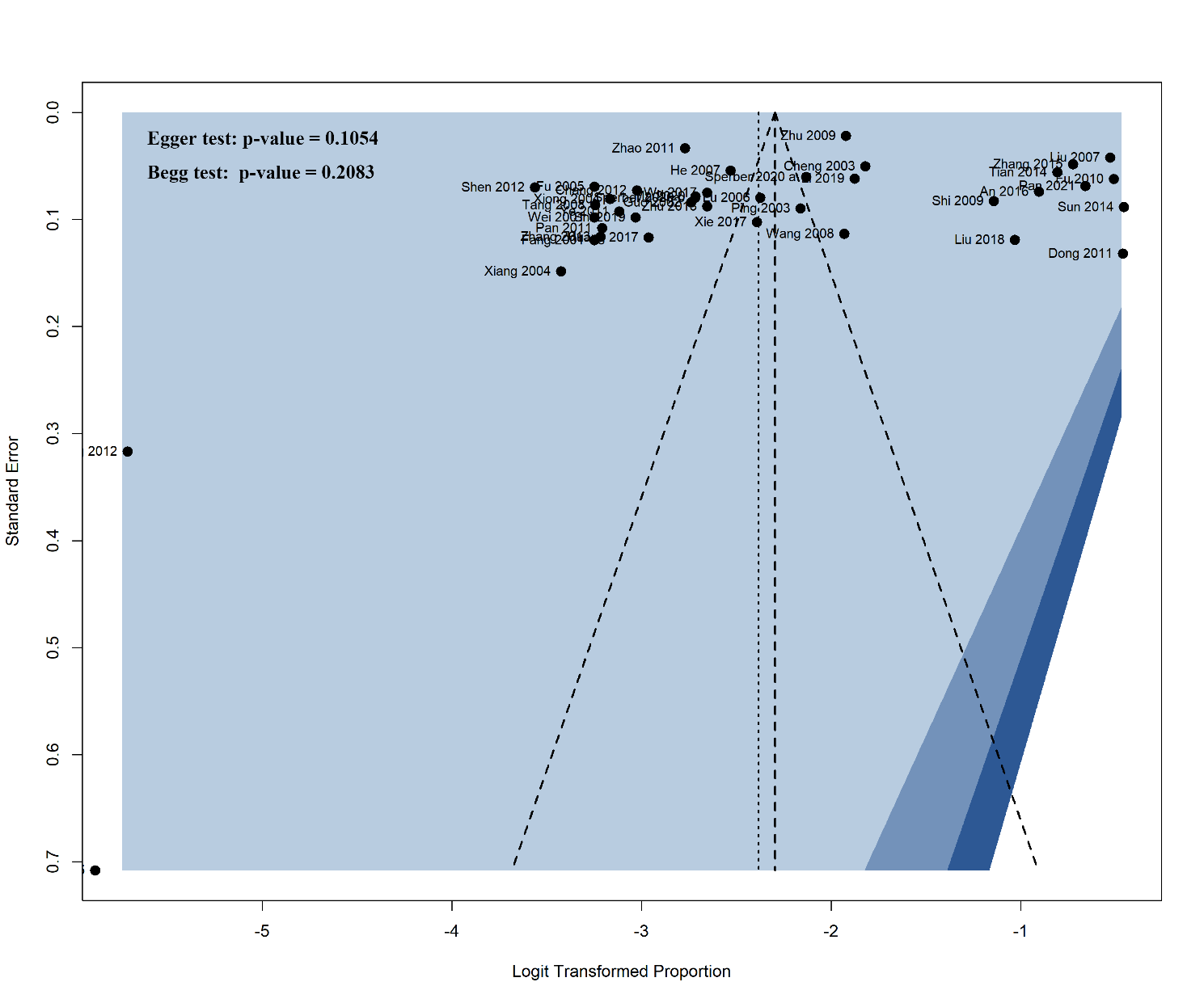


# Supplementary Table 1 Characteristics of included studies

| **Study Year** | **Event** | **Sample size** | **Prevalence** | **Disease type of research** | **Questionnaire method** | **Rome criteria** | **Sampling methods** | **Study year** |
| --- | --- | --- | --- | --- | --- | --- | --- | --- |
| **An 2016** | 257 | 892 | 28.81% | Constipation only | Interview-administered questionnaire | Rome III | Convenience sampling | between 2011-2020 |
| **Chang 2012** | 198 | 4275 | 4.63% | Constipation and other gastrointestinal disorders | Interview-administered questionnaire | Rome III | Stratify and random sampling | between 2001-2010 |
| **Cheng 2003** | 458 | 3282 | 13.95% | Constipation only | Interview-administered questionnaire* | Rome II | Random sampling only | between 2001-2010 |
| **Dong 2011** | 94 | 243 | 38.68% | Constipation only | Unclear administered questionnaire | Rome II | Random sampling only | Unclear |
| **Fang 2001** | 73 | 1952 | 3.74% | Constipation and other gastrointestinal disorders | Interview-administered questionnaire | Rome II | Stratify and random sampling | between 1991-2000 |
| **Fu 2005** | 216 | 5784 | 3.73% | Constipation and other gastrointestinal disorders | Interview-administered questionnaire | Rome II | Random sampling only | between 2001-2010 |
| **Fu 2010** | 413 | 1100 | 37.55% | Constipation only | Interview-administered questionnaire | Rome II | Random sampling only | between 2001-2010 |
| **Guo 2002** | 151 | 2486 | 6.07% | Constipation only | Interview-administered questionnaire | Rome II | Stratify and random sampling | between 1991-2000 |
| **He 2007** | 366 | 4967 | 7.37% | Constipation only | Interview-administered questionnaire | Rome II | Stratify and random sampling | between 2001-2010 |
| **Huang 2017** | 77 | 1568 | 4.91% | Constipation only | Interview-administered questionnaire | Rome III | Stratify and random sampling | between 2011-2020 |
| **Li 2019** | 301 | 2267 | 13.28% | Constipation only | Interview-administered questionnaire | Rome II | Stratify and random sampling | between 2011-2020 |
| **Liu 2007** | 896 | 2414 | 37.12% | Constipation only | Self-administered questionnaire | Rome II | Convenience sampling | between 2001-2010 |
| **Liu 2018** | 96 | 365 | 26.30% | Constipation only | Interview-administered questionnaire | Rome III | Random sampling only | between 2011-2020 |
| **Lu 2006** | 172 | 2018 | 8.52% | Constipation and other gastrointestinal disorders | Interview-administered questionnaire | Rome II | Convenience sampling | between 2001-2010 |
| **Ma 2020** | 173 | 2786 | 6.21% | Constipation only | Interview-administered questionnaire | Rome III | Stratify and random sampling | between 2011-2020 |
| **Pan 2011** | 89 | 2290 | 3.89% | Constipation only | Unclear administered questionnaire | Rome III | Convenience sampling | between 2001-2010 |
| **Pan 2021** | 323 | 947 | 34.11% | Constipation only | Interview-administered questionnaire | Rome III | Random sampling only | between 2011-2020 |
| **Ping 2003** | 139 | 1347 | 10.32% | Constipation and other gastrointestinal disorders | Unclear administered questionnaire | Rome II | Random sampling only | between 1991-2000 |
| **Shen 2012** | 211 | 7648 | 2.76% | Constipation only | Interview-administered questionnaire | Rome III | Stratify and random sampling | between 2001-2010 |
| **Shi 2009** | 192 | 794 | 24.18% | Constipation only | Unclear administered questionnaire | Rome II | Convenience sampling | between 2001-2010 |
| **Shi 2019** | 109 | 2367 | 4.60% | Constipation only | Interview-administered questionnaire | Rome III | Random sampling only | between 2011-2020 |
| **Sperber 2020 a** | 309 | 2914 | 10.60% | Constipation and other gastrointestinal disorders | Self-administered questionnaire* | Rome IV | Convenience sampling | between 2011-2020 |
| **Sperber 2020 b** | 168 | 2710 | 6.20% | Constipation and other gastrointestinal disorders | Interview-administered questionnaire* | Rome IV | Convenience sampling | between 2011-2020 |
| **Sun 2014** | 210 | 541 | 38.82% | Constipation only | Interview-administered questionnaire | Rome III | Random sampling only | between 2011-2020 |
| **Tang 2008** | 139 | 3709 | 3.75% | Constipation only | Interview-administered questionnaire | Rome III | Stratify and random sampling | between 2001-2010 |
| **Tian 2014** | 463 | 1500 | 30.87% | Constipation only | Unclear administered questionnaire | Rome II | Convenience sampling | between 2011-2020 |
| **Wang 2008** | 89 | 703 | 12.66% | Constipation only | Interview-administered questionnaire | Rome II | Convenience sampling | between 2001-2010 |
| **Wang 2012** | 10 | 3032 | 0.33% | Constipation and other gastrointestinal disorders | Interview-administered questionnaire | Rome III | Convenience sampling | between 2011-2020 |
| **Wei 2001** | 108 | 2892 | 3.73% | Constipation and other gastrointestinal disorders | Interview-administered questionnaire | Rome II | Random sampling only | between 1991-2000 |
| **Wu 2017** | 191 | 2906 | 6.57% | Constipation and other gastrointestinal disorders | Self-administered questionnaire | Rome II | Stratify and random sampling | between 2001-2010 |
| **Xiang 2004** | 47 | 1492 | 3.15% | Constipation only | Unclear administered questionnaire | Rome II | Random sampling only | between 2001-2010 |
| **Xie 2017** | 104 | 1241 | 8.38% | Constipation only | Unclear administered questionnaire | Rome III | Stratify and random sampling | between 2011-2020 |
| **Xiong 2004** | 159 | 3931 | 4.04% | Constipation only | Interview-administered questionnaire | Rome II | Stratify and random sampling | between 2001-2010 |
| **Xu 2015** | 2 | 720 | 0.28% | Constipation and other gastrointestinal disorders | Interview-administered questionnaire | Rome III | Convenience sampling | Unclear |
| **Ye 2011** | 122 | 2880 | 4.24% | Constipation only | Unclear administered questionnaire | Rome III | Convenience sampling | between 2001-2010 |
| **Zhang 2013** | 77 | 1999 | 3.85% | Constipation and other gastrointestinal disorders | Interview-administered questionnaire | Rome III | Convenience sampling | Unclear |
| **Zhang 2015** | 634 | 1942 | 32.65% | Constipation only | Interview-administered questionnaire | Rome III | Stratify and random sampling | between 2001-2010 |
| **Zhao 2011** | 948 | 16078 | 5.90% | Constipation and other gastrointestinal disorders | Self-administered questionnaire* | Rome II | Stratify and random sampling | between 2001-2010 |
| **Zhu 2009** | 2425 | 18984 | 12.77% | Constipation only | Self-administered questionnaire | Rome II | Stratify and random sampling | between 2001-2010 |
| **Zhu 2013** | 139 | 2113 | 6.58% | Constipation only | Interview-administered questionnaire | Rome III | Random sampling only | between 2001-2010 |

*: validated questionnaire

# Supplementary Table 2 Pooled prevalence of functional constipation according to dietary habits, living habits, working conditions, and unhealthy behavior

|  | **Number of  studies** | **Number of  participants** | **Pooled prevalence (95% confidence interval)** | **Odds ratio  (95% confidence interval)** | **I²** | **p value  for χ²** |
| --- | --- | --- | --- | --- | --- | --- |
| ***Eating habits*** | | | | | | |
| **High-fiber diet** | | | | | | |
| Take few or never intake | 5 | 1416 | 32.8% (14.5-57.5) | 1 | - | - |
| Take high intake | 5 | 3050 | 14.0% (7.1-25.8) | 0.33 (0.15-0.75) | 93% | ＜0.01 |
| **fruit** | | | | | | |
| Take few or never intake | 4 | 1326 | 24.1% (9.4-49.1) | 1 | - | - |
| Take high intake | 4 | 2288 | 13.6% (5.9-28.3) | 0.51 (0.28-0.92) | 87% | ＜0.01 |
| **vegetable** | | | | | | |
| Take few or never intake | 4 | 1159 | 30.0% (16.1-49.0) | 1 | - | - |
| Take high intake | 4 | 2833 | 14.5% (6.1-30.4) | 0.38 (0.29-0.51) | 36% | 0.19 |
| **Coarse grains** | | | | | | |
| Take few or never intake | 2 | 1589 | 18.5% (7.7-38.1) | 1 | - | - |
| Take high intake | 2 | 2089 | 9.1% (3.2-23.1) | 0.44 (0.35-0.57) | 0% | 0.55 |
| **Meat** | | | | | | |
| Take few or never intake | 2 | 1859 | 8.9% (3.5-20.8) | 1 | - | - |
| Take high intake | 2 | 601 | 22.6% (9.0-46.4) | 2.92 (2.17-3.93) | 0% | 0.81 |
| **Drinking water situation** | | | | | | |
| Low | 2 | 376 | 52.2% (44.6-59.7) | 1 | - | - |
| Moderate | 2 | 870 | 26.7% (17.3-38.9) | 0.34 (0.10-1.14) | 96% | ＜0.01 |
| High | 2 | 432 | 27.8% (21.4-35.1) | 0.35 (0.14-0.87) | 89% | ＜0.01 |
| **Drinking water time** | | | | | | |
| Drinking in the morning | 2 | 518 | 27.2% (11.9-50.9) | 1 | - | - |
| Drinking in the other times | 2 | 1285 | 21.2% (9.2-41.8) | 0.71 (0.57-0.90) | 0% | 0.86 |
| ***living habits*** | | | | | | |
| **Physical activity** | | | | | | |
| Frequent | 9 | 19747 | 9.1% (5.5-14.6) | 1 | - | - |
| Infrequent | 9 | 7472 | 16.7% (8.8-29.3) | 1.97 (1.14-3.43) | 96% | ＜0.01 |
| **Bowel habit** | | | | | | |
| Regular | 5 | 3388 | 15.7% (7.7-29.5) | 1 | - | - |
| Irregular | 5 | 1647 | 41.6% (24.5-61.1) | 3.64 (2.64-5.03) | 70% | 0.01 |
| ***Working*** ***conditions*** | | | | | | |
| **Labor types** | | | | | | |
| Manual | 4 | 117224 | 3.9% (0.9-15.4) | 1 | - | - |
| Mental | 4 | 6876 | 9.7% (3.8-22.5) | 2.66 (0.64-11.08) | 99% | ＜0.01 |
| **Working pressure** | | | | | | |
| Low | 1 | 143 | 11.9% (7.5-18.3) | 1 | - | - |
| High | 1 | 222 | 35.6% (29.6-42.1) | 4.09 (2.3-7.29) | NA | NA |
| **Working status** | | | | | | |
| No working | 2 | 574 | 9.3% (5.2-16.1) | 1 | - | - |
| Working (including Employed and Student and homemaker) | 2 | 4491 | 9.2% (5.2-15.8) | 0.99 (0.74-1.34) | 0% | 0.96 |
| Retired | 2 | 675 | 10.7% (6.5-17.1) | 1.13 (0.78-1.63) | 0% | 0.77 |
| ***Unhealthy behavior*** | | | | | | |
| **Smoking** |  |  |  |  |  |  |
| Never | 7 | 20336 | 8.5% (5.3-13.5) | 1 | - | - |
| Former | 3 | 645 | 10.6% (6.5-16.7) | 1.27 (0.97-1.66) | 0% | 0.52 |
| Current | 6 | 6810 | 8.6% (4.5-16.1) | 0.92 (0.58-1.45) | 92% | ＜0.01 |
| **Alcohol drinking** | | | | | | |
| Drinking | 5 | 4381 | 8.5% (3.7-18.7) | 1 | - | - |
| No drinking | 5 | 18485 | 8.3% (4.5-14.9) | 1.01 (0.62-1.64) | 87% | ＜0.01 |

# Supplementary Table 3 Pooled prevalence of functional constipation according to related diseases

| **Related diseases** | **Number of studies** | **Number of participants (participants with FC/participants without FC)** | **Pooled prevalence in participants with FC  (95% confidence interval)** | **Pooled prevalence in participants without FC  (95% confidence interval)** | **participants with FC vs without FC Odds ratio  (95% confidence interval)** | **I²** | **p value  for χ²** |
| --- | --- | --- | --- | --- | --- | --- | --- |
| **Abnormal mental state** | | | | | | | |
| Anxiety | 5 | 1801/3833 | 20.3% (11.1-34.2) | 6.9% (4.5-10.1) | 3.16 (1.96-5.11) | 81% | ＜0.01 |
| Depression | 5 | 1755/3879 | 20.8% (11.3-35.2) | 8.1% (4.9-13.3) | 2.74 (1.76-4.26) | 78% | ＜0.01 |
| Poor sleep quality | 8 | 1251/17280 | 33.8% (29.2-38.6) | 18.2% (12.9-24.9) | 2.14 (1.69-2.72) | 66% | ＜0.01 |
| **Circulatory system diseases** | | | | | | | |
| Coronary heart disease | 1 | 109/2258 | 20.2% (13.7-29.0) | 17.3% (15.8-18.9) | 1.21 (0.75-1.95) | NA | NA |
| Hypertension | 4 | 659/7507 | 31.5% (24.2-40.0) | 30.6% (19.5-44.6) | 1.11 (0.81-1.52) | 66% | 0.03 |
| **Digestive system diseases** | | | | | | | |
| Biliary tract disease | 3 | 1656/17924 | 3.4% (0.9-12.2) | 2.7% (0.5-14.1) | 1.32 (0.78-2.22) | 48% | 0.15 |
| Dyspepsia | 4 | 1436/21681 | 22.6% (4.7-63.3) | 4.5% (2.4-8.2) | 6.00 (1.48-24.27) | 97% | ＜0.01 |
| Gastroesophageal reflux | 2 | 1099/17465 | 9.9% (4.6-20.2) | 3.9% (2.6-5.9) | 2.69 (1.56-4.66) | 76% | 0.04 |
| **Endocrine system diseases** | | | | | | | |
| Diabetes | 2 | 249/3283 | 13.3% (9.2-18.8) | 9.5% (5.6-15.8) | 1.61 (1.09-2.38) | 0% | 0.53 |
| Hyperlipidemia | 2 | 186/3749 | 15.1% (10.6-20.9) | 17.3% (16.1-18.7) | 0.85 (0.56-1.28) | 0% | 0.59 |

FC: Functional constipation; NA: not applicable, too few studies to assess heterogeneity.

# Supplementary Table 4 Percentage of functional constipation according to self-management behavior

| **Self-management behavior** | **Number of  studies** | **Number of FC patients** | **Percentage (95% confidence interval)** | **I²** | **p value  for x²** |
| --- | --- | --- | --- | --- | --- |
| Health-seeking behavior | 11 | 1862 | 17.2% (11.5-24.8) | 92% | ＜0.01 |
| Change in nothing | 4 | 820 | 23.4% (14.8-34.8) | 94% | ＜0.01 |
| Self-medicine behavior | 4 | 820 | 23.8% (13.9-37.5) | 93% | ＜0.01 |
| Change in diets | 4 | 808 | 40.7% (29.9-52.6) | 94% | ＜0.01 |

FC: Functional constipation.

# Supplementary File 1 The Detailed Search Strategy

**The Pubmed database Search Strategy**

#1: ((((((((constipation[MeSH Terms]) OR (functional constipation[MeSH Terms])) OR (constipation[Title/Abstract])) OR (functional constipation[Title/Abstract])) OR (chronic functional constipation[Title/Abstract])) OR (idiopathic constipation[Title/Abstract])) OR (chronic idiopathic constipation[Title/Abstract])) OR (bowel disorders[Title/Abstract])) OR (functional gastrointestinal disorders[Title/Abstract])

Results 33197

#2: (((((((((Rome) OR (Rome I)) OR (Rome 1)) OR (Rome II)) OR (Rome 2)) OR (Rome III)) OR (Rome 3)) OR (Rome IV)) OR (Rome 4)

Results 153582

#3: (((((((((((((((((((((((((((((((((((((China[Title/Abstract]) OR (Chinese[Title/Abstract])) OR (China)) OR (Chinese)) OR (Beijing)) OR (Hebei)) OR (Inner Mongolia Autonomous Region)) OR (Shanxi)) OR (Tianjin)) OR (Heilongjiang)) OR (Jilin)) OR (Liaoning)) OR (Anhui)) OR (Fujian)) OR (Jiangsu)) OR (Jiangxi)) OR (Shandong)) OR (Shanghai)) OR (Zhejiang)) OR (Guangdong)) OR (Guangxi Zhuang Autonomous Region)) OR (Hainan)) OR (Henan)) OR (Hubei)) OR (Hunan)) OR (Chongqing)) OR (Guizhou)) OR (Sichuan)) OR (Tibet Autonomous Region)) OR (Yunnan)) OR (Gansu)) OR (Ningxia Hui Autonomous Region)) OR (Qinghai)) OR (Shaanxi)) OR (Xinjiang Uyghur Autonomous Region)) OR (Hongkong)) OR (Macau)) OR (Taiwan)

Results 2551722

#4：#1 AND #2 AND #3

Results 176

**The Cochrane library database Search Strategy**

#1: (constipation OR functional constipation OR chronic functional constipation OR idiopathic constipation OR chronic idiopathic constipation OR bowel disorders OR functional gastrointestinal disorders):ti,ab,kw

Results 14432

#2: (Rome OR Rome I OR Rome 1 OR Rome II OR Rome 2 OR Rome III OR Rome 3 OR Rome IV OR Rome 4):ti,ab,kw

Results 2616

#3: (China OR Chinese OR Beijing OR Hebei OR Inner Mongolia Autonomous Region OR Shanxi OR Tianjin OR Heilongjiang OR Jilin OR Liaoning OR Anhui OR Fujian OR Jiangsu OR Jiangxi OR Shandong OR Shanghai OR Zhejiang OR Guangdong OR Guangxi Zhuang Autonomous Region OR Hainan OR Henan OR Hubei OR Hunan OR Chongqing OR Guizhou OR Sichuan OR Tibet Autonomous Region OR Yunnan OR Gansu OR Ningxia Hui Autonomous Region OR Qinghai OR Shaanxi OR Xinjiang Uyghur Autonomous Region OR Hongkong OR Macau OR Taiwan)

Results 106328

#:4：#1 AND #2 AND#3

Results 65

**The Embase database Search Strategy**

#1: (constipation:ab,ti OR 'functional constipation':ab,ti OR 'chronic functional constipation':ab,ti OR 'idiopathic constipation':ab,ti OR 'chronic idiopathic constipation':ab,ti OR 'bowel disorders':ab,ti OR 'functional gastrointestinal disorders':ab,ti)

Results 50134

#2: (rome:ab,ti OR 'rome i':ab,ti OR 'rome 1':ab,ti OR 'rome ii':ab,ti OR 'rome 2':ab,ti OR 'rome iii':ab,ti OR 'rome 3':ab,ti OR 'rome iv':ab,ti OR 'rome 4':ab,ti)

Results 17643

#3: China OR Chinese OR Beijing OR Hebei OR Inner Mongolia Autonomous Region OR Shanxi OR Tianjin OR Heilongjiang OR Jilin OR Liaoning OR Anhui OR Fujian OR Jiangsu OR Jiangxi OR Shandong OR Shanghai OR Zhejiang OR Guangdong OR Guangxi Zhuang Autonomous Region OR Hainan OR Henan OR Hubei OR Hunan OR Chongqing OR Guizhou OR Sichuan OR Tibet Autonomous Region OR Yunnan OR Gansu OR Ningxia Hui Autonomous Region OR Qinghai OR Shaanxi OR Xinjiang Uyghur Autonomous Region OR Hongkong OR Macau OR Taiwan

Results 351660

#4: #1 AND #2 AND #3

Results 39

**Web of Science**

#1: (constipation OR functional constipation OR constipation OR functional constipation OR chronic functional constipation OR idiopathic constipation OR chronic idiopathic constipation OR bowel disorders OR functional gastrointestinal disorders):ti,ab,kw

Results 48802

#2: (Rome OR Rome I OR Rome 1 OR Rome II OR Rome 2 OR Rome III OR Rome 3 OR Rome IV OR Rome 4):ti,ab,kw

Results 37562

#3: (China OR Chinese OR Beijing OR Hebei OR Inner Mongolia Autonomous Region OR Shanxi OR Tianjin OR Heilongjiang OR Jilin OR Liaoning OR Anhui OR Fujian OR Jiangsu OR Jiangxi OR Shandong OR Shanghai OR Zhejiang OR Guangdong OR Guangxi Zhuang Autonomous Region OR Hainan OR Henan OR Hubei OR Hunan OR Chongqing OR Guizhou OR Sichuan OR Tibet Autonomous Region OR Yunnan OR Gansu OR Ningxia Hui Autonomous Region OR Qinghai OR Shaanxi OR Xinjiang Uyghur Autonomous Region OR Hongkong OR Macau OR Taiwan):ti,ab,kw

Results 7468251

#4: #1 AND #2 AND #3

Results 208
